# Evoked potentials in the Atlantic cod following putatively innocuous and putatively noxious electrical stimulation: a minimally invasive approach

**DOI:** 10.1007/s10695-013-9834-2

**Published:** 2013-07-30

**Authors:** Stian Ludvigsen, Niels C. Stenklev, Helge K. Johnsen, Einar Laukli, Dagfinn Matre, Øyvind Aas-Hansen

**Affiliations:** 1 Department of Arctic and Marine Biology, Faculty of Biosciences, Fisheries and Economics, University of Tromsø, 9037 Tromsö, Norway; 2 Faculty of Health Sciences, Institute of Clinical Medicine, University of Tromsø, 9037 Tromsö, Norway; 3 Department of Work Psychology and Physiology, National Institute of Occupational Health, 0033 Oslo, Norway; 4 Norwegian Institute of Food, Fisheries and Aquaculture Research (Nofima), Muninbakken 9-13, P.O. Box 6122, 9291 Breivika, Norway; 5 Present Address: Faculty of Health Sciences, Institute of Medical Biology, University of Tromsø, 9037 Tromsö, Norway

**Keywords:** Somatosensory evoked potentials, Nociception, Pain, Teleost fish, Brain, EEG

## Abstract

Aspects of peripheral and central nociception have previously been studied through recording of somatosensory evoked potentials (SEPs) to putative noxious stimuli in specific brain regions in a few freshwater fish species. In the present study, we describe a novel, minimally invasive method for recording SEPs from the central nervous system of the Atlantic cod (*Gadus morhua*). Cutaneous electric stimulation of the tail in 15 fish elicited SEPs at all stimulus intensities (2, 5, 10 and 20 mA) with quantitative properties corresponding to stimulus intensity. In contrast to previous fish studies, the methodological approach used in Atlantic cod in the current study uncovered a number of additional responses that could originate from multiple brain regions. Several of these responses were specific to stimulation at the highest stimulus intensities, possibly representing qualitative differences in central processing between somatosensory and nociceptive stimuli.

## Introduction

The question of nociception and a possible capacity for pain perception in fish represents a topical and highly controversial issue (Braithwaite and Huntingford 2004; Chandroo et al. 2004; Huntingford et al. 2006; Rose 2002; Rose 2007; Rose et al. 2012; Sneddon 2011). In humans, pain perception consists of two associated, but distinct components; nociception and pain (Loeser and Treede 2008). The former is prevalent in the animal kingdom and concerns the ability to detect harmful (noxious) stimuli, which requires an appropriate sensory apparatus (Kavaliers 1988; Smith and Lewin 2009). Pain, however, includes not only a sensory component but is also a psychological state which includes an unpleasant emotional experience (IASP 1979; Loeser and Treede 2008; Merskey et al. 1994). Whereas nociceptive processing may occur, unconsciously, in both lower and higher regions of the central nervous system (CNS), pain perception requires mental awareness (consciousness) which presuppose a highly developed brain (Brooks and Tracey 2005; Treede et al. 1999).

Given the impossibility of asking an animal whether it feels pain, one criterion put forward when assessing nociception and the potential for pain perception in animals is that there has to be a pathway from nociceptors in the periphery to higher brain regions (Bateson 1992; Dunstan et al. 1991). One way to map such a pathway is to record somatosensory evoked potentials (SEPs). SEPs are weak electric responses in the CNS following stimulation of peripheral sensory nerves. Evaluation of SEPs is an important tool in research on nociception and pain in mammals (Kakigi et al. 2000, 2005). In a few species of freshwater fishes [goldfish (*Carassius auratus*), rainbow trout (*Oncorhynchus mykiss*) and Atlantic salmon (*Salmo salar*)], it has previously been demonstrated that putatively non-noxious and noxious stimulation elicited SEPs in different brain regions including the telencephalon (Dunlop and Laming 2005; Nordgreen et al. 2007).

To proceed further in the debate on nociception and potential pain perception in fish, more knowledge on central nociceptive processing is needed. In the present study, the main aim was to present a novel, minimally invasive approach to assess evoked potentials with putatively nociceptive stimuli in fish, and also to investigate whether we could reproduce findings in freshwater species on a marine teleost fish, the Atlantic cod (*Gadus morhua*). Our minimally invasive method was adapted from studies of auditory evoked potentials in fish (Faucher et al. 2009; Kenyon et al. 1998) and from studies of evoked potentials with nociception and potential pain in human infants [e.g., (Slater et al. 2010a, b, c)]. Briefly, this method involves temporal summation of repetitive stimulus-locked recordings, but rather than using intracranial electrodes in spatially designated brain areas the evoked potentials are recorded from the EEG of the whole brain by using subcutaneous electrodes.

## Experimental procedures

### Subjects

Fifteen artificially reared Atlantic cod (*G. morhua*) measuring 30-38 cm were used in these experiments. The fish were transported from the Tromsø Aquaculture Research Station in Kårvik to the University of Tromsø and held in large, aerated seawater holding tanks connected to a flow-through system (water temperature 7 °C), at least 5 days before initiation of experiments. The fish were not fed during this time. Experiments were performed in accordance with the guidelines of the Norwegian Animal Welfare Act (National Animal Research Authority of Norway, application approval number 11462011), which adheres to the European Convention for the Protection of Vertebrate Animals used for Experimentation and other Scientific Purposes (Council of Europe 1998).

### Experimental preparations

**Fig. 1:**
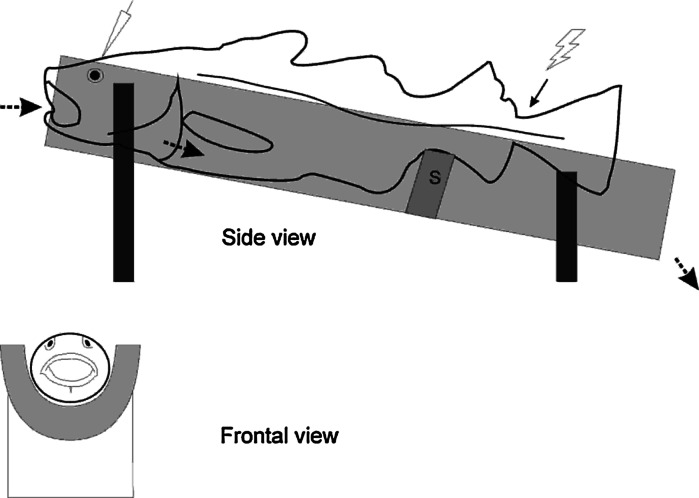
The custom-built fish cradle consisted of a PVC-pipe cut in half and tilted at an angle so that the seawater/anesthetic could exit the cradle by gravitation (flow direction indicated by *dotted arrows*). *S* supports bracket that kept the fish fairly level and ensured that the tail and the stimulating electrodes (denoted by *lightning bolt* and *arrow*) were kept dry. Positioning of the recording electrodes is indicated on top of the head. Figure also shows frontal view the cradle

Prior to the experiments, the fish were transferred from the holding tank to a 10-L bucket and anaesthetized in seawater containing 10 mg/L Aquacalm (Metomidate, Syndel International Inc., Qualicum Beach, Canada). After approximately 5 min, when the fish did no longer respond to a pinch at the base of the tail (Horsberg 1994), it was removed from the bucket, and immediately given an injection of 0.08 mg/100 g body weight Pavulon (Pancuronium bromide 2 mg/ml, Schering-Plough AB, Stockholm, Sweden) in the caudal vessels using a 1-mL syringe. Pavulon was administered to minimize muscular twitching during experiments. It was then transferred to a purpose-built cradle (Fig. 1) and restrained loosely with metal strips (room temperature 17.5 °C). The skin was protected from the metal strips by a layer of moist cloth covered by an additional layer of aluminum foil. A silicon tube was carefully placed in the mouth of the fish once restrained in order to administer a continuous flow of oxygen-saturated seawater (7 °C) with maintenance anesthesia (3 mg/L Aquacalm) over the gills. When this seawater exited the gills, the flow continued ventrally along the belly of the fish to the back of the cradle (which was tilted downwards) and left the cradle via a draining tube. That is, the areas of the skin intended for electrode placement were kept dry to avoid shunting of the current by seawater. The seawater/anesthesia was delivered from a 100-L reservoir, and adequate flow (1 L/min) was assured by a flow valve. The fish was observed for at least 30 min after the recording and stimulating electrodes had been positioned, to ensure that the injected Pavulon had taken effect.

### Electrodes and recording equipment

Insulated stainless steel recording electrodes (Bio-logic Systems Corp., Mundelein, IL, USA) with only the tip exposed (length 5 mm, diameter 1 mm) were placed subcutaneously between the eyes, along the mid-sagittal plane of the fish, so that the recording electrodes were positioned above the telencephalon. The two electrodes were positioned in the midline, 10 mm apart, while the ground electrode was positioned close, but slightly anterior to the foremost recording electrode. The signal was amplified ×100000 using a CP122 AC/DC strain gage amplifier (Grass Instrument Co., Warwick, RI, USA) and filtered through a 50-Hz band-stop filter with high pass (1 Hz) and low pass (1000 Hz), before being digitized (sampling frequency 1600 Hz) and fed into a computer. AEP software version 6.1.0 (Bio-logic Systems Corp., Mundelein, IL, USA) was used for acquisition and analysis of data. Impedance was measured before each experiment and was always below 3 kΩ.

Two blunt, stainless steel electrodes glued to a strip of Velcro (35 mm apart) were used for cutaneous stimulation. The Velcro was attached around the base of the tail with the two electrodes positioned laterally so that one was above the lateral line on the right side, and the other was below the lateral line on the left side. Care was taken to avoid contact of the electrodes with the lateral line. The distance between stimulating and recording electrodes was on average 19.2 cm. Stimulus trains consisting of 30 rectangular electrical pulses with a duration of 1 ms and an inter-stimulus interval of 3 s were given by a custom-built stimulator at each intensity (2, 5, 10 and 20 mA) to all animals. The stimulator was triggered by a Navigator^®^PRO (Bio-logic Systems Corp., Mundelein, IL, USA) preamplifier controlled by the AEP software. Responses were recorded in a 320-ms time window from the stimulus onset, with the duration of the recordings and the number of stimuli being set from preceding pilot experiments. For the pilot experiments, the time window used was 0-1000 ms, but this was shortened to 320 ms for subsequent recordings as there were no responses beyond that. During the pilot phase of the study, postmortem stimulation with accompanying recordings was performed to exclude the presence of electromagnetic artifacts. The fish were immediately euthanized after the experiment with a blow to the head.

### Data analysis and statistics

Weighted grand means of the recorded responses at respective stimulus intensities were plotted to identify activity peaks (both positive and negative) using the AEP software. Latencies for maximal peak amplitudes were identified using time cursors. We then identified and found latencies of peaks in each individual experiment (i.e., averages of the 30 stimulations at each of the respective amplitudes in each fish) that most likely corresponded to those of the weighted means. Peak-to-peak amplitudes were measured manually in printouts and converted to μV. To assess whether peak latencies, peak-to-peak amplitude and peak duration (width in ms) changed over the four different stimulus intensities, within-subject differences were compared using repeated measures analysis of variance (RM-ANOVA) or the Wilcoxon matched pairs rank sum test. Only data for peaks with responses at all four levels of stimuli were analyzed. All statistics were performed using SPSS 19 (SPSS Inc., Armonk, NY, USA).

## Results

**Fig. 2:**
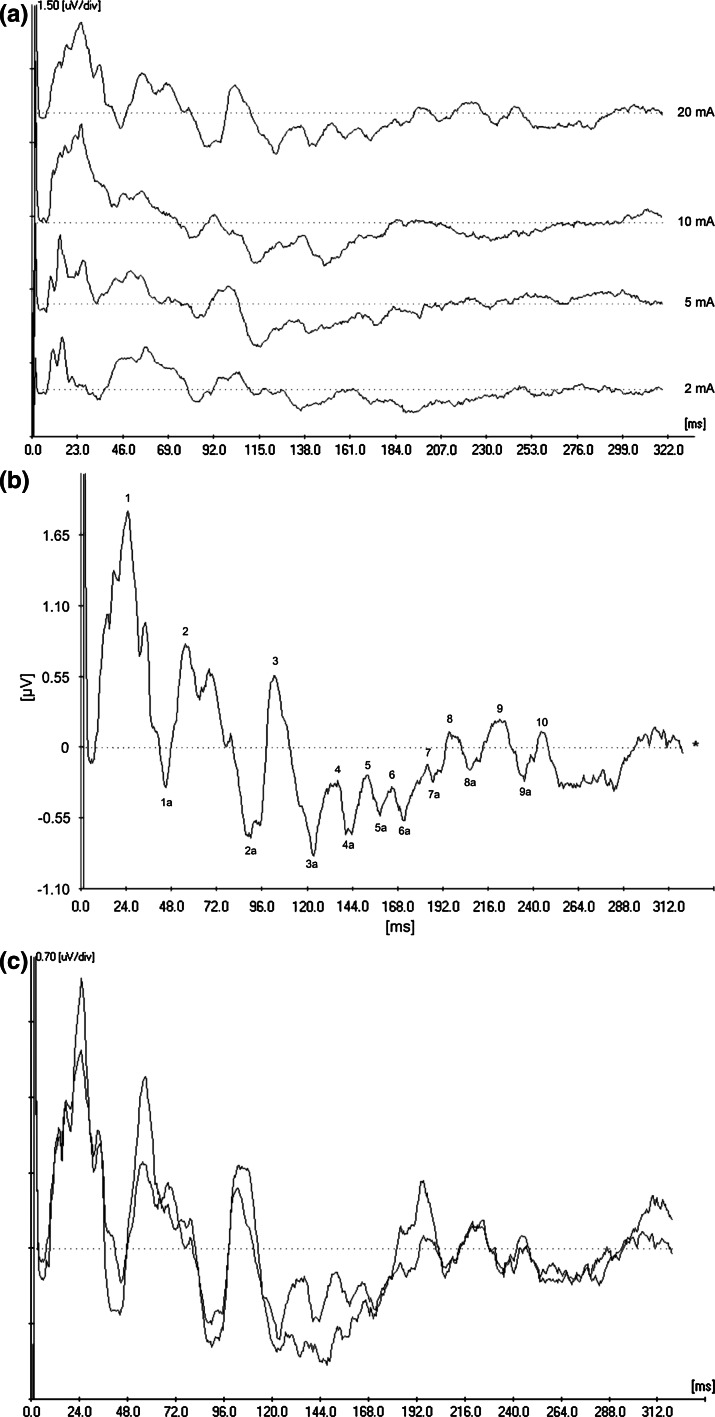
Somatosensory evoked potentials (SEPs) in response to cutaneous electrical stimulation at the base of the tail in Atlantic cod. **a** Weighted average (inverted) of responses to stimulation with currents of 2, 5, 10 and 20 mA (bottom to top). *X*-axis shows time in ms while the scale on the *y*-axis is 1.50 μV/div. *Dotted lines* are baselines. **b** Weighted average (inverted) of responses to stimulation with 20 mA. *Numbers* denote the identified activity peaks. Peaks 1–4a and peaks 6, 6a and 7 were identified in responses from all four stimulus intensities. Peak 7a was identified only in responses to stimulation with 10 and 20 mA, and peaks 5, 5a, 8, 8a and 10 were identified in responses to stimulation with 20 mA only. *X*-axis shows time in ms while y-axis shows μV. *Dotted line* is the baseline. **c** Weighted average (inverted) of 50 % of recorded responses to 20-mA stimulation superimposed on the weighted average of *all* recorded responses to 20-mA stimulation. The high degree of conformity was interpreted as a validation that peaks exclusively seen in response to 20-mA stimulation were indeed biological activity. *X*-axis shows time in ms while the scale on the *y*-axis is 0.70 μV/div

Cutaneous stimulation at the base of the tail in anesthetized Atlantic cod elicited SEPs in all animals. The responses consisted of several consecutive peaks appearing up to a maximum of 250-ms post-stimulation (Fig. 2a). Peaks within the first 160 ms [peak 1–4a (Fig. 2b)] appeared in recordings from all stimulus intensities. Between 160 and 250 ms, we identified up to eleven additional peaks [peak 5–10 (Fig. 2b)] of which three (peak 6, 6a and 7) were found in recordings from all stimulation intensities, two (peak 7a and 9) were found only in recordings following stimulation with 10 and 20 mA and the remaining five (peak 5, 5a, 8, 8a and 10) appeared only in recordings following stimulation with 20 mA. In order to verify peaks that appeared only at the stronger stimulus amplitudes (i.e., 10 and 20 mA), grand means of 50 % of the recorded responses (randomly selected) from the respective stimulus amplitudes were superimposed on the grand mean of *all* recorded responses from the same amplitude, as shown in Fig. 2c for 20 mA. A good conformity of the grand means was interpreted as verification that peaks represented true biological activity, and not artifacts. Six recordings were excluded from the results for magnetic interference. Furthermore, a given peak did not always appear in every recording, resulting in some variation in the number of animals included in the respective groups.

RM-ANOVA analysis showed a significant change in latency to maximum peak for peaks 1, 1a, 3, 3a, 6, 6a and 7 (Table 1). The assumption of sphericity was fulfilled for all analyzed data regarding peak latency. *Post hoc* analysis (Bonferroni) revealed correlation between stimulus amplitude and peak latency for peaks 1a, 3 and 6 (Table 1). Latencies to maximum peak in relation to increasing fish length (i.e., increased distance between stimulating and recording electrodes) showed a positive relationship for some, but not all, fish.

**Table 1:** Summary of statistics on peak latencies

Peak	Peak latency (mean) (ms)	*n*	Sphericity^1^	*F*-value^2^	Within-subject effect^2^	Pairwise comparison^3^
1			*p* = 0.25	2,99	*p* = 0.05	No significance
2 mA	16.67	10				
5 mA	16.12	10				
10 mA	18.18	10				
20 mA	20.80	10				
1a			*p* = 0.07	4,41	*p* = 0.01	2 vs. 20 mA:
2 mA	32.08	10				*p* = 0.015
5 mA	32.92	10				
10 mA	35.67	10				
20 mA	39.91	10				
3			*p* = 0.93	6,58	*p* = 0.002	10 vs. 20 mA:
2 mA	101.38	10				*p* = 0.03
5 mA	100.06	10				
10 mA	92.11	10				
20 mA	104.68	10				
3a			*p* = 0.60	3,27	*p* = 0.04	No significance
2 mA	116.04	9				
5 mA	117.98	9				
10 mA	110.90	9				
20 mA	123.32	9				
6			*p* = 0.49	14,45	*p* = 0.00	2 vs. 5, 10,
2 mA	159.27	7				20 mA:
5 mA	168.29	7				*p* = 0.02
10 mA	167.40	7				*p* = 0.037
20 mA	176.50	7				*p* = 0.02
6a			*p* = 0.30	3,89	*p* = 0.03	No significance
2 mA	171.41	7				
5 mA	175.25	7				
10 mA	172.84	7				
20 mA	181.85	7				

Shown are only values for peaks with significant differences

^1^Mauchley’s test

^2^RM-ANOVA

^3^Bonferroni

There was a positive correlation between peak-to-peak amplitude and stimulus amplitude for several peaks, but none of these correlations were found to be statistically significant in the RM-ANOVA test. As average values for peak-to peak amplitude for peak 1 and 1a showed an evident correlation with stimulus amplitude, we performed the nonparametric Wilcoxon matched pairs rank sum test on these data. We then found statistically significant differences in peak-to-peak amplitude between stimulation with 2 and 20 mA for both peaks (*p* = 0.03 for both).

**Fig. 3:**
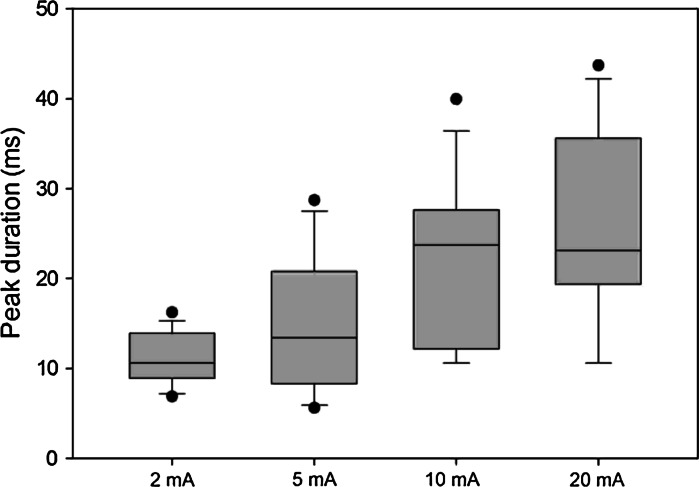
Box plot showing duration (ms) of peak 1 following stimulation with 2, 5, 10 and 20 mA, respectively. *Horizontal lines inside boxes* represent medians, box edges represent 80 % confidence interval, bars represent 5th/95th percentiles and *filled circles* represent outliers. Peak duration increased with increasing stimulus intensity

We also noted that the width (i.e., duration in ms) of peak 1 increased as stimulus amplitude increased (Fig. 3). This increase was statistically significant (*n* = 10, *F* = 8.14, *p* = 0.001), and according to post hoc analysis, the differences in duration between responses from 2 mA (10.99 ms) and 10 mA (22.99 ms), and 2 and 20 mA (22.93 ms) were significant (*p* = 0.01 for both comparisons).

## Discussion

This study demonstrated that cutaneous electric stimulation at the base of the tail elicited somatosensory evoked responses in the central nervous system of the Atlantic cod. In contrast to previous work in goldfish (*C. auratus*), rainbow trout (*O. mykiss*) (Dunlop and Laming 2005) and the Atlantic salmon (*S. salar*) (Nordgreen et al. 2007), this was achieved using a minimally invasive approach to study responses from the entire brain including the brainstem (Faucher et al. 2009; Kenyon et al. 1998; Slater et al. 2010c). Whereas previous studies in other fish species found SEPs with a maximum of two or three response categories and with a maximum latency of about 70 ms, the present approach identified up to 19 (positive and negative) different response peaks with latencies up to 250 ms. The stimulus intensities used in this study were identical to those used in the Atlantic salmon (Nordgreen et al. 2007) which were based on stimulus intensities used in comparable studies on humans and rats (Chen and Herrmann 2001; Stienen et al. 2003). While Nordgreen et al. (2007) suggested that stimulus intensities considered aversive for mammals may also be aversive to fish on the basis of the general organization of the fish nervous system (Balment et al. 1998) and the characteristics of fish nociceptors (Ashley et al. 2007; Sneddon 2003), others argue that this is highly unlikely since fish do not possess a neocortex, a structure considered imperative for pain perception in mammals (Rose et al. 2012). Although experimental studies suggest that electric stimulation may induce aversive behaviors in fish [e.g., Chervova (1997), Dunlop et al. (2006), Ehrensing et al. (1982)], a direct comparison of stimulus intensities with those studies is not possible since they report stimulus intensities in volts with no information on impedance. There is considerable variation in reported pain thresholds for humans [see, e.g., Chen and Herrmann (2001) and Sang et al. (2003)], and whether a transcutaneous electrical stimulus is painful depends not only on current amplitude, but also on current density [size of the electrode, Inui et al. (2003)]. Accordingly, in the present study, reservations must be made when interpreting the results as we do not know whether the stimulus intensities used in the present study truly activated nociceptive afferents or would result in aversive behavioral responses in an awake fish. However, as we to some degree found positive correlations between response amplitudes and stimulus intensity as previously reported by other workers (Chen and Herrmann 2001; Nordgreen et al. 2007; Stienen et al. 2003), we considered 10 and 20 mA to be putatively noxious. Under this assumption, the longer latency responses seen only at 10 and 20 mA (i.e., peak 7a at 191–197 ms) or only at 20 mA (i.e., peaks 5, 5a, 8, 8a, 9a and 10, from 158 to 243 ms) may represent central responses that are specific to nociceptive stimuli.

When recording SEPs in the telencephalon of Atlantic salmon following presumed innocuous and increasingly noxious levels of electric stimulation to the tail (Nordgreen et al. 2007), a negative peak with a latency of about 30 ms was seen in all individuals and at all stimulus intensities and a secondary negative peak with a latency of about 74 ms was seen in some individuals predominantly at higher stimulus intensities. Based on estimates of conduction velocities and the observed latencies, it was suggested that the first peak depended on the activation of peripheral A-delta fibers whereas the second peak depended on the activation of peripheral C-fibers (Nordgreen et al. 2007). Similarly, when recording evoked responses from different brain areas of goldfish and rainbow trout following putative innocuous and noxious mechanical stimulation of the skin, two or three response categories were detected and suggested to depend on the activation of A-delta and C-fibers based on conduction velocity measurements (Dunlop and Laming 2005). In the present study, the application of the same estimates would suggest that peak latencies in the present study correspond to the findings in Atlantic salmon, rainbow trout and goldfish, implying that the majority of the recorded responses may comply with modality-specific early components originating from the activation of A-delta fibers and C-fibers. The short latency peak 1 may represent far-field potential originating from the brainstem (Faucher et al. 2009; Liberson 1994; Polak et al. 2009) or activation of A-beta fibers (but evidence from investigations in Atlantic salmon, goldfish and rainbow trout (Dunlop and Laming 2005; Nordgreen et al. 2007; Sneddon 2003) suggests this fiber type to be of little significance to measured responses in fish). Furthermore, as our recordings were not specific to a single brain region, the additional peaks observed in this study may corroborate a previous study in the rainbow trout and goldfish (Dunlop and Laming 2005) which recorded SEPs in different parts of the fish brain. It should be noted, however, that although A-delta mechanoreceptors as well as A-delta and C-fiber nociceptors have been demonstrated in the rainbow trout (Ashley et al. 2007; Sneddon 2002, 2003; Sneddon et al. 2003), this has not yet been specifically investigated in the Atlantic cod. It should also be noted that although the presence of C-fibers in other teleost fish has been demonstrated histologically, their numbers are very low compared to humans (Roques et al. 2010; Sneddon 2002).

In this study, the stimuli were presented close to the lateral line of the fish, and as such there is a chance that activity in the posterior lateral line nerve (PLL) could have been picked up in the recordings and misinterpreted as part of the SEPs. Under the assumption that the PLL of the Atlantic cod has similar properties as that of the rainbow trout (Schellart and Kroese 2002), peaks of evoked potentials originating from the lateral line would have latencies of approximately 8 ms in the telencephalon [using the average conduction velocity of 22.9 m s^−1^ (Schellart and Kroese 2002)]. As all but one of the registered peaks had maximum peak latencies longer than 10 ms, we consider it unlikely that activity in the PLL may have confounded our results.

In contrast to previous studies in fish, the present study investigated SEPs in a longer time window to identify possible later responses (0–1000 ms during pilot experiments). In humans, late responses are considered to be involved in non-specific multimodal processing of somatosensory stimuli but are sometimes also found to differ between innocuous and noxious stimuli (Niedermeyer and Lopes DaSilva 2005; Slater et al. 2010a, b, c). It is difficult to interpret whether the later responses observed exclusively for the highest stimulus amplitudes (10 and 20 mA) in Atlantic cod in the present study are equivalents to such late responses in mammals, but this finding is interesting, and should be explored in future studies.

In conclusion, this study has demonstrated that SEPs following peripheral electrical stimulation can be readily recorded using a minimally invasive approach with subcutaneous recording electrodes. Compared to previous studies recording SEPs from specific brain regions, we recorded several additional responses, possibly originating from multiple brain regions. The recorded responses had quantitative properties that correlated with stimulus amplitude. The minimally invasive method shows promise within future research on nociception in fish, as regulations concerning the use of animals in experimental research are getting ever stricter. There are still many stones to be turned before the potential for nociception and potential pain perception in fish and other animals has been fully clarified.
